# Infection with novel coronavirus (SARS-CoV-2) causes pneumonia in *Rhesus macaques*

**DOI:** 10.1038/s41422-020-0364-z

**Published:** 2020-07-07

**Authors:** Chao Shan, Yan-Feng Yao, Xing-Lou Yang, Yi-Wu Zhou, Ge Gao, Yun Peng, Lian Yang, Xue Hu, Jin Xiong, Ren-Di Jiang, Hua-Jun Zhang, Xiao-Xiao Gao, Cheng Peng, Juan Min, Ying Chen, Hao-Rui Si, Jia Wu, Peng Zhou, Yan-Yi Wang, Hong-Ping Wei, Wei Pang, Zheng-Fei Hu, Long-Bao Lv, Yong-Tang Zheng, Zheng-Li Shi, Zhi-Ming Yuan

**Affiliations:** 1 Center for Biosafety Mega-Science, Wuhan Institute of Virology, Chinese Academy of Sciences, Wuhan, Hubei 430071 China; 2 State Key Laboratory of Virology, Chinese Academy of Sciences, Wuhan, Hubei 430071 China; 3 Department of Forensic Medicine, Tongji Medical College of Huazhong University of Science and Technology, 13 Hangkong Road, Wuhan, Hubei 430074 China; 4 Department of Radiology, Union Hospital, Tongji Medical College, Huazhong University of Science and Technology, Wuhan, Hubei 430022 China; 5 University of Chinese Academy of Sciences, Beijing, China; 6 Key Laboratory of Animal Models and Human Disease Mechanisms of the Chinese Academy of Sciences, Kunming Institute of Zoology, Chinese Academy of Sciences, Kunming, Yunnan 650223 China; 7 Kunming Primate Research Center of the Chinese Academy of Sciences, Kunming Institute of Zoology, Chinese Academy of Sciences, Kunming, Yunnan 650223 China

**Keywords:** Whole genome amplification, Genome-wide association studies

## Abstract

The 2019 novel coronavirus (SARS-CoV-2) outbreak is a major challenge for public health. SARS-CoV-2 infection in human has a broad clinical spectrum ranging from mild to severe cases, with a mortality rate of ~6.4% worldwide (based on World Health Organization daily situation report). However, the dynamics of viral infection, replication and shedding are poorly understood. Here, we show that *Rhesus macaques* are susceptible to the infection by SARS-CoV-2. After intratracheal inoculation, the first peak of viral RNA was observed in oropharyngeal swabs one day post infection (1 d.p.i.), mainly from the input of the inoculation, while the second peak occurred at 5 d.p.i., which reflected on-site replication in the respiratory tract. Histopathological observation shows that SARS-CoV-2 infection can cause interstitial pneumonia in animals, characterized by hyperemia and edema, and infiltration of monocytes and lymphocytes in alveoli. We also identified SARS-CoV-2 RNA in respiratory tract tissues, including trachea, bronchus and lung; and viruses were also re-isolated from oropharyngeal swabs, bronchus and lung, respectively. Furthermore, we demonstrated that neutralizing antibodies generated from the primary infection could protect the *Rhesus macaques* from a second-round challenge by SARS-CoV-2. The non-human primate model that we established here provides a valuable platform to study SARS-CoV-2 pathogenesis and to evaluate candidate vaccines and therapeutics.

## Introduction

Coronavirus infections are common in humans and other mammals. Coronaviruses belong to the family Coronaviridae and possess a single-stranded, positive-sense RNA genome ranging from 26 to 32 kilobases in length. In December 2019, a cluster of cases of pneumonia were reported in people associated with the Huanan Seafood Wholesale Market in Wuhan, Hubei Province, China. The subsequent research from the bronchoalveolar lavage fluid (BALF) of a hospitalized patient allowed the isolation of a novel coronavirus, which was named severe acute respiratory syndrome coronavirus 2 (SARS-CoV-2) and considered as the pathogen of the current outbreak of coronavirus disease (COVID-19).^1^ The common clinical features for COVID-19 include fever, cough, myalgia, fatigue, dyspnea, lymphopenia, and pneumonia; less common symptoms include sputum production, headache, haemoptysis, and diarrhea.^2^ As of May 26, 2020, a total of 5,370,375 cases had been reported in 216 countries, areas or territories with more than 344,454 deaths,^3^ whereas a sizable portion of infected but non-symptomatic people with potential of transmissibility was also reported.^4,5^ Due to the urgency of the situation, World Health Organization declared COVID-19 as a global pandemic on March 11, 2020.

Animal models are essential for the study of pathogenesis of the viral infection, the evaluation of potential antiviral treatments or vaccine development. Here we report a nonhuman primate disease model for SARS-CoV-2. We experimentally infected *Rhesus macaques* (RMs) using SARS-CoV-2 isolation from clinical BALF,^6^ and investigated the dynamic changes of SARS-CoV-2 in blood, swabs and respiratory tract tissues and the histopathological changes of lungs in infected RMs. The results indicated that SARS-CoV-2 caused the interstitial pneumonia and bronchopneumonia in lungs.

## Results

### SARS-CoV-2 replication dynamics in RMs

**Fig. 1:**
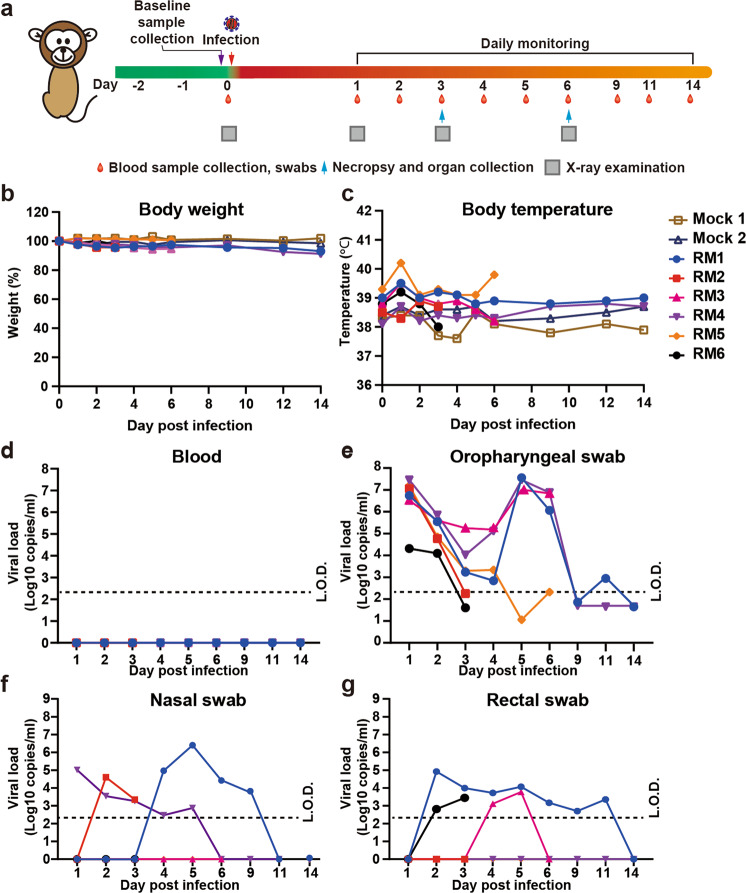
Experimental scheme, body weight change, body temperature change and viral RNA load dynamics in SARS-CoV-2-infected RMs. **a** Experimental scheme and experimental parameters. Six RMs were intratracheally inoculated with 7 × 10^6^ TCID_50_ of SARS-CoV-2. Mock 1 and Mock 2 were two control animals treated with DMEM. Disease parameters were measured including changes of body weight, body temperature and behaviors at the indicated time points. Viral loads in blood and swabs were monitored to evaluate viral replication kinetics in RMs. Blood and swab sample collection, chest X-ray examination and necropsy with organ pathological examination were performed at the indicated time points. The viral RNA was extracted by Qiagen Viral RNA kit, followed by RT-qPCR to quantify viral RNA. **b** Body weight changes of RMs after infection with SARS-CoV-2. **c** Changes of rectal temperature of RMs after infection with SARS-CoV-2. **d** Viral RNA load in blood. **e** Viral RNA load in oropharyngeal swabs. **f** Viral RNA load in nose. **g** Viral RNA load in rectal tract. L.O.D. limit of detection, 200 copies/mL.

Six RMs (1–6) between the ages of 6 and 11 years (Supplementary information, Table S1), three males and 3 females, were inoculated with 7 × 10^6^ 50% tissue-culture infectious doses (TCID_50_) of SARS-CoV-2 (Institute of Virology, Chinese Academy of Sciences, IVCAS 6.7512) and 2 RMs were inoculated with Dulbecco’s modified Eagle’s medium (DMEM) through intratracheal route. The blood, oropharyngeal swab, nasal swab and rectal swab were evaluated post infection (Fig. 1a). No obvious clinical signs were observed during the study course except that one animal showed reduced appetite. All animals investigated did not show body weight changes from 1 to 6 days post infection (d.p.i.), while 7% and 8% weight loss was observed at 14 d.p.i. in RM1 and RM4, respectively (Fig. 1b). Body temperatures were monitored from day 1 to day 14 and no obvious changes were found (Fig. 1c).

Hematologic analysis was performed for blood collected during clinical exams (Supplementary information, Fig. S1). Overall, the number of white blood cells decreased after infection, whereas the number of neutrophils decreased in all animals by 2 d.p.i. and was maintained at a stable level until 6 d.p.i., and the number of lymphocytes increased from day 1 to day 6. However, the importance of the hematologic changes and their relationship with the viral infection still need to be further elaborated.

To examine the viral replication dynamics in RM, blood, oropharyngeal swab, nasal swab and rectal swab were collected at 1–6, 9, 11 and 14 d.p.i. from the RMs available for study. No viral RNA could be detected in blood from day 1 to day 14 by quantitative reverse transcriptase polymerase chain reaction (RT-qPCR) (Fig. 1d). Two peaks of viral RNA load were observed in oropharyngeal swabs from most animals at 1 and 5 d.p.i. (Fig. 1e). The first peak on day 1 was observed in oropharyngeal swabs from all six animals (RM1-RM6) and viral RNA levels ranged from 2.08 × 10^4^ copies/mL to 2.85 × 10^7^ copies/mL. Due to the euthanization of two animals on day 3 and another two on day 6 for pathological examination, the animal sample size for the following investigation was reduced to 4 on day 4 and 2 after day 6 (see below). Notably, a second peak of viral load in oropharyngeal swab appeared on day 5 after infection with viral loads of 1.03 × 10^7^–3.60 × 10^7^ copies/mL in three out of the four animals investigated (RM1, RM3 and RM4), whereas one monkey (RM5) that exhibited a very low viral RNA load (<10 copies/mL) on day 5 showed an increased viral load on day 6 after infection (~200 copies/mL). The viral RNA levels in oropharyngeal swabs fell rapidly after day 6 in the two remaining RMs (RM1 and RM4) and the viral RNA reached undetectable level in these two animals by day 9. The nasal swabs also started to show viral RNA-positive signal in 3 (RM1, RM2 and RM4) out of 6 RMs at 1, 2 or 4 d.p.i. and remained positive for viral RNA for 2–5 days (Fig. 1f). The positive signal reflected the viral RNA in nasal cavity which could be brought from the lower respiratory tract by breathing. Furthermore, we evaluated the rectal viral RNA from 1 to 14 d.p.i. (Fig. 1g). Three out of six RMs exhibited detectable viral RNA levels on day 2 or day 4 with RM1 showing the longest duration of viral shedding from day 2 till day 11. Detection of viral RNA in rectal swab may indicate potential infection in digestive tract. The possible process might be that the virus co-infected both the trachea and the esophagus, and then the viral RNA was detected in rectal swab after virus went through the digestive tract. To confirm whether infectious viruses exist in those swabs, TCID_50_ assay was performed for oropharyngeal swab, nasal swab and rectal swab samples on Vero cells (Supplementary information, Fig. S2). The infectious viral load showed the same pattern as viral RNA load. On day 1, the viral load in oropharyngeal swabs ranged from 1.40 × 10^4^ to 1.40 × 10^6^ TCID_50_/mL, while the second peak showed up at 5 d.p.i. with viral load ranging from 7.14 × 10^3^ to 1.32 × 10^4^ TCID_50_/mL (Supplementary information, Fig. S2a). For nasal swabs, the infectious virus was only found in RM1 at 5 d.p.i., and no virus was found in rectal swabs (Supplementary information, Fig. S2b, c). Collectively, the viral RNA and infectious viruses were detected in upper respiratory tract after infection. The two peaks in oropharyngeal swabs could represent the original input and viral replication in the respiratory tract, respectively.

### Respiratory tract injury in SARS-CoV-2-infected animal

**Fig. 2:**
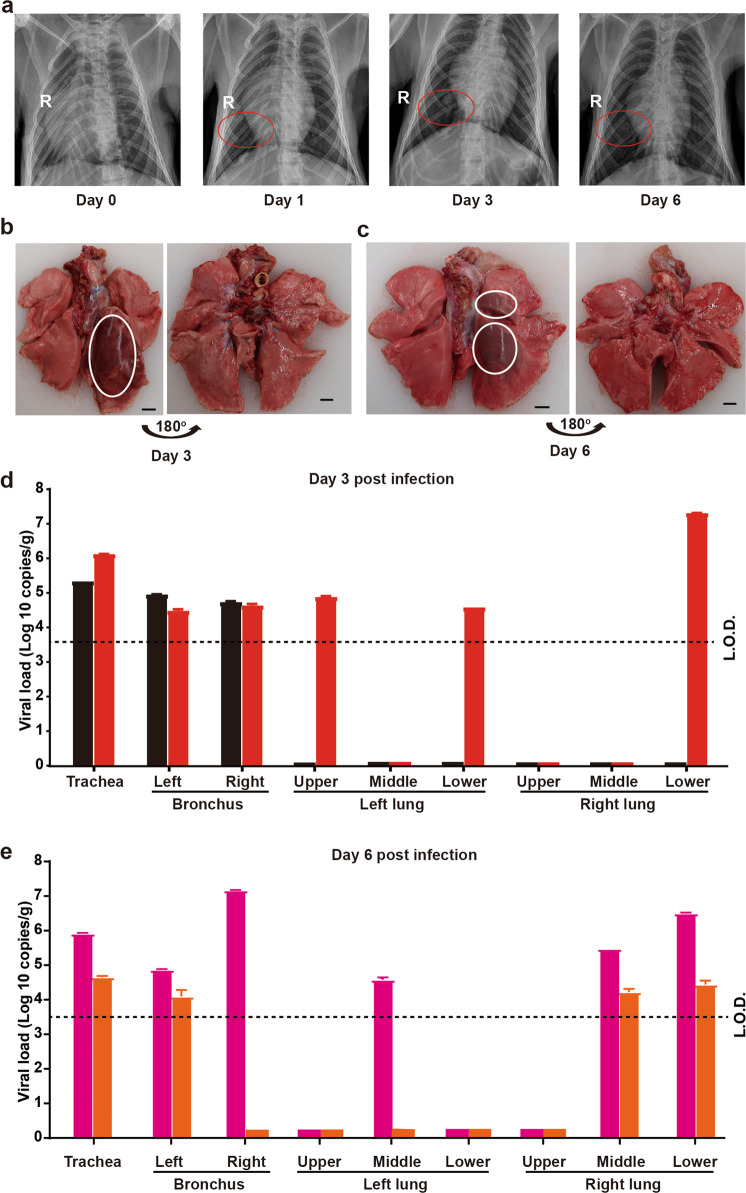
Characterization of the respiratory tract changes after SARS-CoV-2 infection. **a** Radiographs of the chest of RM before inoculation and on day 3 and day 6 post infection with SARS-CoV-2. The red circled areas are regions of interstitial infiltrates indicative of viral pneumonia. **b**, **c** Lesions in the lungs. A view of the ventral lungs of an infected animal obtained at necropsy on day 3 and 6 post infection, showing severe hemorrhage and necrosis of lung (white circles). Scale bar, 5 mm. Viral loads in trachea, bronchus, right and left upper, middle, and lower lung lobes on day 3 (**d**) and day 6 (**e**). Red bar, RM2; black bar, RM6; pink bar, RM3; orange bar, RM5. Error bars mean standard deviation from two technical repeats. L.O.D. limit of detection, 10^3^–10^4^ copies/g as the tissue weight varies.

The chest X-ray examination was conducted in animals at 0, 1, 3 and 6 d.p.i. As shown in Fig. 2a (left panel), normal lungs were presented before infection, while patchy glass-ground opacity was observed in the lower parts of right lungs at 1 d.p.i. (Fig. 2a, middle left panel). At 3 d.p.i., multiple glass-ground opacities in right lungs were found (Fig. 2a, middle right panel) and the density decreased slightly at 6 d.p.i. (Fig. 2a, right panel), while the scope of lung lesions extended from day 3 to day 6. Two animals (RM2 and RM6) were euthanized at 3 d.p.i., while another two (RM3 and RM5) were euthanized at 6 d.p.i. The two remaining animals were housed until the secondary infection. Necropsy was then performed in all the four animals. Postmortem examinations showed a variable degree of consolidation, edema, hemorrhage and necrosis throughout the lower respiratory tract, indicating the interstitial pneumonia. The section of the inferior lobe of the right lung is dark red with large patchy hemorrhage accounting for ~15% of the lung tissue on day 3, and the animal had two red dark foci in the right upper lobe and right lower lobe accounting for ~10% of the lung tissue on day 6 (Fig. 2b, c). However, the surface and section of the lungs were gray and white with consolidation, and the inferior lobes of lungs were firmer than the upper lobes in dark red with patchy hemorrhage. After necropsy, the tissues and organs were harvested for quantification of viral RNA. RT-qPCR analysis was performed for both respiratory tract tissues and other organs. The data revealed the widespread presence of SARS-CoV-2 in the respiratory tract. The amount of the viral RNA ranged from 3.0 × 10^4^ to 1.5 × 10^7^ copies/g in trachea and bronchus tract at both 3 (Fig. 2d) and 6 d.p.i. (Fig. 2e). In the lungs, viral RNA was detected with titer up to 2.0 × 10^7^ copies/g and mainly localized in the lower lobes of lungs, but no specific viral RNA could be detected in heart, liver, spleen, kidney, intestine, stomach and reproductive tract on day 3 and 6 post infection (Supplementary information, Fig. S3). Taken together, we presume that the respiratory tract is the preliminary target of SARS-CoV-2 infection, while other organs might not be the direct targets.

### Histopathological changes in rhesus macaques infected with SARS-CoV-2

**Fig. 3:**
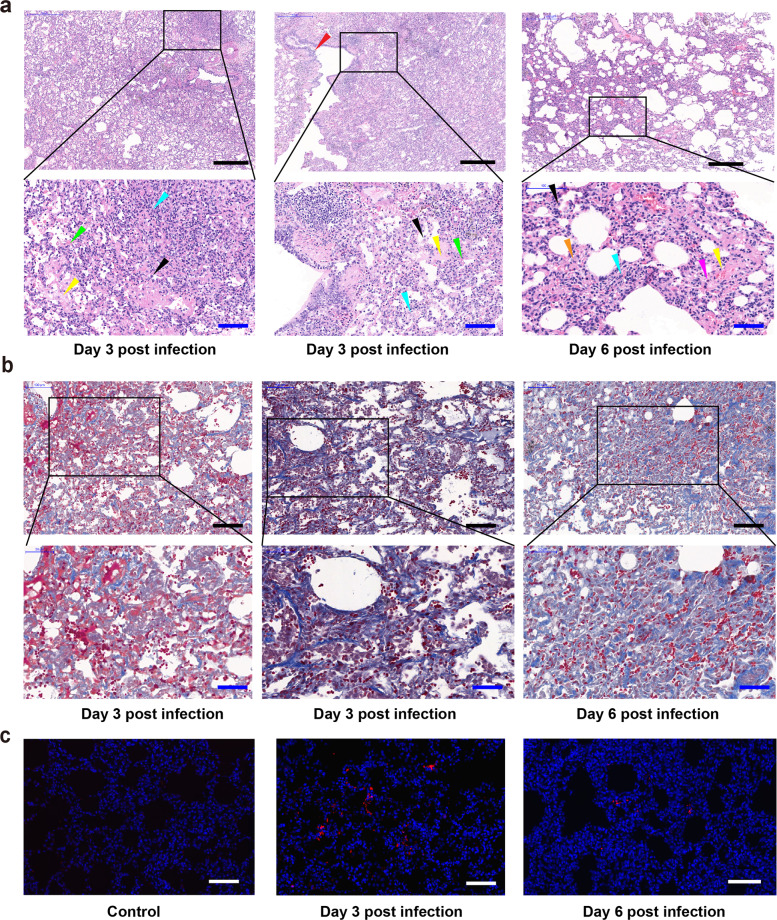
Histopathological analysis of lung changes in RMs infected with SARS-CoV-2. Six RMs were inoculated with SARS-CoV-2. Two animals were euthanized at 3 days post infection, and 2 animals at 6 days post infection. Histological analysis was performed on tissues collected at 3 and 6 days post infection. **a** HE staining of the lung tissues. **b** Masson staining of lung tissue. **c** Immunofluorescence analysis of SARS-CoV-2 in lung tissue. A rabbit polyclonal antibody raised against the SARS-CoV NP protein was used for specific staining of SARS-CoV-2 antigen. Black scale bar, 500 µm; blue scale bar, 50 µm; white scale bar, 50 µm. Blue triangle, pulmonary fibrosis; black triangle, macrophages; yellow triangle, edema; green triangle, pulmonary hyaline membrane formation; pink triangle, neutrophil.

Upon necropsy, the area of lung lobes affected by lesions was estimated by pathologists. Histopathological analysis showed injury surrounding small bronchus, with the most injury in the inferior lobe of the left and right lungs. Three days after infection, SARS-CoV-2 caused interstitial pneumonia in the RMs. Thickened alveolar walls were observed, with infiltrations of a large number of monocytes and lymphocytes, and a few eosinophils. In the severe lesion area, alveolar wall necrosis, collapse and fibrosis can be seen, accompanied by extensive fibroblast proliferation. In alveolar cavities, a slight and moderate cellulose exudation can be commonly observed, and the hyaline membrane formation and pulmonary hemorrhage were observed in some of them (Fig. 3a, b). Meanwhile, the epithelial cells of the bronchioles were necrotic and exfoliated, mononuclear and lymphocytic infiltrations were observed in the mucosa and submucosal layer, and even peribronchitis occurred. There is clear thrombus in pulmonary capillary lumen, with endothelial cell swelling and perivasculitis (Fig. 3a, b). In contrast, no histopathological changes were found in control animals (Supplementary information, Fig. S4).

After 6 days of infection, the lung lesions of RMs were alleviated, with decrease of monocyte and lymphocyte infiltrations, increase of macrophages, and reduction of edema and hyaline membrane. The obvious fibrosis and organization of alveolar wall and alveolar cavity were revealed, showing a “glomerular” nodular hyperplasia. There is compensatory emphysema in a portion of alveoli.

Confocal microscopy analysis of lesion sites using specific antibodies (anti-CoV nucleoprotein (N) protein) indicated the positive viral N protein (Fig. 3c). Besides the interstitial pneumonia and bronchopneumonia in lung, obvious edema of tracheal and bronchial epithelial cells, loss of cilia in some of them, and infiltrations of monocytes, lymphocytes and a small number of eosinophils in lamina propria and submucosa were also observed on day 3 post infection (Supplementary information, Fig. S5). On day 6 post infection, the mucosal lesions of trachea and bronchus were significantly alleviated and the epithelial cell structure was basically restored, but a small number of monocytes, lymphocytes and eosinophils were still seen in the submucosa. The histopathological changes of heart, liver, kidney, spleen, stomach, ileum, colon and testis were shown in Supplementary information, Fig. S6. Those organs showed varying levels of edema, increase or infiltration of inflammatory cells on day 3 post infection, while the symptom was relieved slightly on day 6 post infection. These results suggested that SARS-CoV-2 could infect the respiratory tract resulting in interstitial pneumonia in RMs.

To confirm that the disease was caused by SARS-CoV-2, the virus was re-isolated from bronchus, lung tissues and oropharyngeal swab. The supernatant from homogenized organ/tissue was used to infect Vero cells. The supernatant from cell culture was harvested once the cytopathic effect (CPE, cells get round and dead cells float in wells) was observed (Supplementary information, Fig. S7). The RNA was subsequently extracted from the supernatant and used for further whole-genome sequencing. The whole-genome sequence alignment analysis showed that the re- isolated viruses were >99.99% identical to the original input viral samples (Table 1).

**Table 1:** The genome sequencing results of re-isolated virus from tissues.

Sample	Start	End	Match	Identity (%)
Reference sequence	1	29,891	–	–
Throat	1	29,867	29,854	>99.99
Lung	1	29,867	29,854	>99.99
Bronchus	1	29,878	29.871	>99.99

Reference sequence: GeneBank ID MN996528.1.

### SARS-CoV-2 infection against re-challenge in RMs

To examine whether neutralizing antibody was generated after infection, the sera from RM1 and RM4 were used for plaque reduction neutralization test (PRNT). As shown in Table 2, no neutralizing antibody could be detected in RM1 and RM4 at 7 d.p.i., and then the neutralizing antibody titers in RM1 increased to 1:1350 at 14 d.p.i. and 1:4050 at 21 d.p.i. and RM4 maintained the same titer above 1:12,150 at both 14 and 21 d.p.i.

**Table 2:** The neutralizing antibody titers in sera from SARS-CoV-2-infected RMs.

Macaque identification#	7 d.p.i.	14 d.p.i.	21 d.p.i.
RM1	<50	1:1350	1:4050
RM4	<50	1:>12,150	1:>12,150

**Fig. 4:**
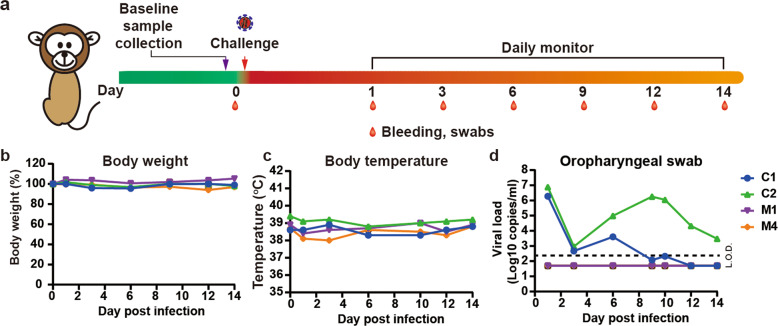
Challenge on the infected RMs. **a** Experimental scheme and experimental parameters. Two pre-infected and two control (C1 and C2) RMs were intratracheally inoculated with 1 × 10^6^ TCID_50_ of SARS-CoV-2. Disease parameters were measured including changes of body weight, body temperature and behaviors at the indicated time points. Viral loads in blood and swabs were monitored to evaluate viral replication kinetics in RMs. Body weight (**b**) and body temperature (**c**) were monitored at the indicated time points. **d** Viral load from oropharyngeal swabs were monitored by qRT-PCR. L.O.D. limit of detection, 200 copies/mL.

To investigate whether the neutralizing antibody can provide protection from SARS-CoV-2 infection, RM1, RM4 and another 2 RMs (C1 and C2) were used for secondary infection (Fig. 4a). Based on our dose response results (data not published), 1 × 10^6^ TCID_50_ of virus was used in secondary infection. RM1, RM4, C1 and C2 were inoculated with 1 × 10^6^ TCID_50_ of SARS-CoV-2 through intratracheal route. The swab samples were collected on days 1, 3, 6, 9, 10, 12 and 14 post infection. No obvious changes of body weight and body temperature during study course were observed. The viral RNA from oropharyngeal swabs showed that the virus still maintained the similar replication pattern in control groups (C1 and C2) to that in Fig. 1; the viral load varied between 1.9 × 10^6^ and 7.7 × 10^6^ copies/mL on day 1 post infection and reached the second peak with 4.0 × 10^3^ and 1.8 × 10^6^ copies/mL on day 6 and day 9 post infection, respectively. In contrast, no viral RNA could be detected in RM1 and RM4, which indicated that the neutralizing antibody might provide protection from the secondary infection.

## Discussion

Since the outbreak, establishing the animal model for SARS-CoV-2 to assess the efficacy of medical countermeasures has been ongoing. To date, a couple of animal models have been built such as non-human primate, transgenic mice as well as ferrets. Two groups showed that the viral RNA could be excreted from nose and throat in the absence of clinical signs in SARS-CoV-2-infected macaques, and pulmonary infiltrates and histological lesions could also be observed.^7,8^ By using transgenic mice bearing human ACE2, Shi and Qin groups found that the infected mice generated typical interstitial pneumonia and pathology that were similar to those of COVID-19 patients.^9–11^ Ferrets were also used as a potential animal model. Earlier studies showed that ferrets were permissive to the SARA-CoV-2 infection and could be transmitted by direct contact.^12,13^ The Yen group investigated the golden Syrian hamsters as an infection model. The data showed that viral RNA was continuously detected in the nasal washes of inoculated hamsters for 14 days and SARS-CoV-2 was transmitted efficiently from inoculated hamsters to naïve hamsters by direct contact and via aerosols.^14^ The clinical symptoms for COVID-19 patients range from asymptomatic to mild to fatal.^15–17^ Although we observed lung injury after infection from our animal model, no obvious clinical signs were captured in our study course which was in agreement with published studies.^7,18^ A retrospective study in China showed that compared to the recovered group, more patients in the death group exhibited pre-existing comorbidities, dyspnea and decrease of oxygen saturation, characteristics of advanced age.^19^ In another study investigated in China, 1099 COVID-19 cases showed that majority of the cases (84.3%) were non-severe, ~5% of confirmed cases required ICU attendance after severe pneumonia occurred, 2.3% needed mechanical ventilation support, and 1.4% died.^5^ The RM model described here exhibits the asymptomatic and mild symptoms, which is in line with the majority of non-severe human cases, although it lacks clinical signs. Interestingly, we observed two peaks of the viral RNA from oropharyngeal swabs, which is also in agreement with other reports.^7,18^ The first peak of the viral RNA should come from the input of the virus, whereas the second peak was due to the authentic viral replication on site. In human, the shedding of SARS-CoV-2 viral RNA was observed in the upper and lower respiratory tract and occasionally in the intestinal tract, similar to our finding in RMs.^15^ This animal model has confirmed the causal relationship between SARS-CoV-2 and respiratory disease in RM reminiscent of the mild respiratory symptom or non-symptomatic cases in COVID-19 already reported in humans.^4^ Interestingly, the data showed less virus and mild inflammation in lung of day 6 (Fig. 3), while there is high viral titer in oropharyngeal swab. Similarly, more virus and severe inflammation were observed in lung on day 3 (Fig. 3) with a low viral RNA titer in oropharyngeal swab. The possible reason is that the newly generated virus was released over time. The virus replicated on site in respiratory tract from day 1 to 3 post infection while there was not too much virus released at this time; the injury happened due to the on-site replication. Newly synthesized virus started to be released to the respiratory tract after day 3 and the peak titer was observed in oropharyngeal swab. However, the inflammation in lung was in the recovery stage after day 3 and less damage was observed. The neutralizing antibody generated by infection could prevent secondary infection by SARS-CoV-2, which suggested that humoral immunity could play a role in protection, although T cell and innate immune could be further investigated in the future to examine their role in the protection. Taken together, in this RM model, SARS-CoV-2 could replicate and be shed throughout the respiratory tract, including oropharyngeal, nasal cavity and alveoli. Replication and shedding in the upper respiratory tract reflect the transmission between hosts, while pulmonary infiltrates and histological lesions may be mainly due to the on-site replication in lower respiratory tract. The established model enables detailed studies of the pathogenesis of this disease and may play a critical role in the evaluation of therapeutic drugs and vaccines. However, the more detailed histopathological changes caused by SARS-CoV-2 in respiratory track and other organs, as well as their relationship with viral infection still awaits investigation.

## Materials and methods

### Ethics statement

All animal experiments were approved by the Institutional Animal Care and Use Committee (Ethics number: WIVA42202001) of Wuhan Institute of Virology, Chinese Academy of Sciences. The SARS-CoV-2 animal model experiments and protocols were also discussed explicitly and extensively with biosafety officers and facility managers at the Wuhan Institute of Virology. All experiments were conducted within the animal biosafety level 4 (ABSL-4) facility in National Biosafety Laboratory (Wuhan), Chinese Academy of Sciences.

### Virus, cell and antibody

SARS-CoV-2 (IVCAS 6.7512) was isolated from BALF collected from a patient with viral pneumonia in December of 2019 in Wuhan, China. The virus isolation was propagated in Vero E6 cells (ATCC® CRL-1586™) in DMEM supplemented with 10% fetal bovine serum (FBS), 1 mM l-glutamine, 100 international units (IU)/mL penicillin and 100 µg/mL streptomycin, and cultured at 37 °C in 5% CO_2_. The virus was harvested on day 3 post infection. The anti-SARS-CoV-2 NP protein antibody was made in house. Cy3-conjugated goat anti-rabbit IgG was purchased from Abcam.

### Routine hematology analysis

The routine hematology was determined from EDTA blood with the IDEXX ProCyte DX analyzer (IDEXX Laboratories) following the instructions of the manufacturer.

### Virus titration

Virus titrations were performed by endpoint titration in Vero E6 cells. Cells were inoculated with 10-fold serial dilutions of cell supernatant. One hour after inoculation of cells, the inoculum was removed and replaced with 100 μL of DMEM supplemented with 2% FBS, 1 mM l-glutamine, 100 IU/mL penicillin, and 100 μg/mL streptomycin. Three days after inoculation, CPE was scored and the TCID_50_ was calculated.

### Animal study and sample collection

Six RMs (three males, three females; 6–11 years of age, 5–12 kg weight), were inoculated with SARS-CoV-2 (7 × 10^6^ TCID_50_) diluted in DMEM. The RMs were anesthetized, and 1 mL of the inoculum was administered intratracheally. Two mock RMs were intratracheally inoculated with DMEM to serve as controls. The RMs were observed twice daily, with detailed recording of clinical signs, symptoms, morbidity, and mortality, including the nature, onset, severity, and duration of all gross or visible changes. After anesthetic treatment, the animals were transferred and X-ray examinations were performed at 0, 1, 3 and 6 d.p.i. by HF100Ha (MIKASA, Japan) for all the available RMs. Swab samples of the oropharyngeal, nasal turbinate, and rectal regions were collected at 1–6, 9 and 14 d.p.i. Swabs were placed into 1 mL of DMEM supplemented with 100 IU/mL penicillin and 100 μg/mL streptomycin. Whole blood was collected in K_2_EDTA tubes for viral RNA extraction and analysis. To confirm the pathogenesis and injury in the respiratory tract, two infected RMs were sacrificed at 3 d.p.i. (RM2 and RM6) and 6 d.p.i. (RM3 and RM5), respectively. The trachea, right bronchus, left bronchus, all six lung lobes and other tissue organs were collected on the day of euthanization for various pathological, virological, and immunological analyses.

For the secondary infection, RMs were inoculated with SARS-CoV-2 (1 × 10^6^ TCID_50_) diluted in DMEM. The swab samples of the oropharyngeal, nasal turbinate, and rectal regions were collected at 1, 3, 6, 9, 12 and 14 d.p.i. The body temperature and body weight were measured at the same time point when sampling.

### RT-PCR

Viral RNA in the samples was quantified by one-step real-time quantitative RT-PCR.^6^ The swab and blood samples were used to extract viral RNA by using the QIAamp Viral RNA Mini Kit (Qiagen), according to the manufacturer’s instructions. Tissues were homogenized in DMEM (1:10, W/V), the homogenate was clarified by low-speed centrifugation at 4500 × *g* for 30 min at 4 °C and the supernatant was immediately used for RNA extraction. RNA was eluted in 50 μL of elution buffer and used as the template for RT-PCR. The primer pairs targeting S gene were used following our previous study: RBD-qF1: 5′-CAATGGTTTAACAGGCACAGG-3′; RBD-qR1: 5′-CTCAAGTGTCTGTGGATCACG-3′. Two microliters of RNA were used to verify the RNA quantity by HiScript® II One Step qRT-PCR SYBR® Green Kit (Vazyme Biotech Co., Ltd) according to the manufacturer’s instructions. The amplification was performed as follows: 50 °C for 3 min, 95 °C for 30 s followed by 40 cycles (95 °C for 10 s, 60 °C for 30 s), with a default melting curve step in an ABI stepone machine.

### Histopathology and immunohistochemistry

Animal necropsies were performed according to a standard protocol. Tissues for histological examination were stored in 10% neutral-buffered formalin for 7 days, paraffin-embedded, sectioned, and stained with hematoxylin and eosin prior to examination by light microscopy. To examine the SARS-CoV-2 antigen, paraffin dehydrated tissue sections were placed in antigen repair buffer for antigen retrieval in a microwave oven. The tissue was blocked with 5% BSA at room temperature for 1 h, followed by incubation with house-made primary antibody at 1:500 (rabbit anti-CoV N protein polyclonal antibody). After being washed by PBS, the slices were slightly dried and covered with Cy3-conjugated goat anti-rabbit IgG (Abcam) at 1:200 dilution. The slides were stained with DAPI (5 µg/mL) after washing by PBS. The image was collected by Pannoramic MIDI system (3DHISTECH, Budapest, Hungary).

### Virus re-isolation and sequencing

The supernatant from homogenized samples was used for re-isolating the virus. Three hundred microliters of supernatant was used to infect the monolayer Vero cells in a 24-well plate. After 1 h inoculation, the supernatant was removed and replaced with fresh DMEM containing 2% FBS plus penicillin and streptomycin. CPE was monitored daily. The supernatant was subjected to viral RNA extraction once CPE was observed. The next generation sequencing was performed as previously reported.^6^

### Neutralizing antibody titer

The virus neutralization test was performed in a 12-well plate. The serum samples from RMs were heat-inactivated at 56 °C for 30 min. The serum samples were diluted at 1:50, 1:150, 1:450, 1:1350, 1:4050 and 1:12,150, and then an equal volume of virus stock was added and incubated at 37 °C in a 5% CO_2_ incubator. After 1 h incubation, 100 µL mixtures were inoculated onto monolayer Vero cells in a 12-well plate for 1 h with shaking every 15 min. The inocula were removed and cells were incubated with DMEM supplemented with 2% FBS containing 0.9% methylcellulose for 3 days before fixation. The cells were then fixed with 4% formaldehyde for 30 min. The formaldehyde solution was removed and the cells were washed by tap water, followed by crystal violet staining. The plaques were counted for calculating the titer.

## Supplementary information

Supplementary Figure S1

Supplementary Figure S2

Supplementary Figure S3

Supplementary Figure S4

Supplementary Figure S5

Supplementary Figure S6

Supplementary Figure S7

Supplementary Table S1

## Supplementary information

**Supplementary information** accompanies for this paper at 10.1038/s41422-020-0364-z.
