# Supplementary material for: Infection with novel coronavirus (SARS-CoV-2) causes pneumonia in Rhesus macaques
Source: Cell Res. 2020 Jul 7;30(8):670–7. doi: 10.1038/s41422-020-0364-z (PMC7364749; doi:10.1038/s41422-020-0364-z)
Supplement: Supplementary file 1 — Supplementary Figure S1 [file 41422_2020_364_MOESM1_ESM.pdf]

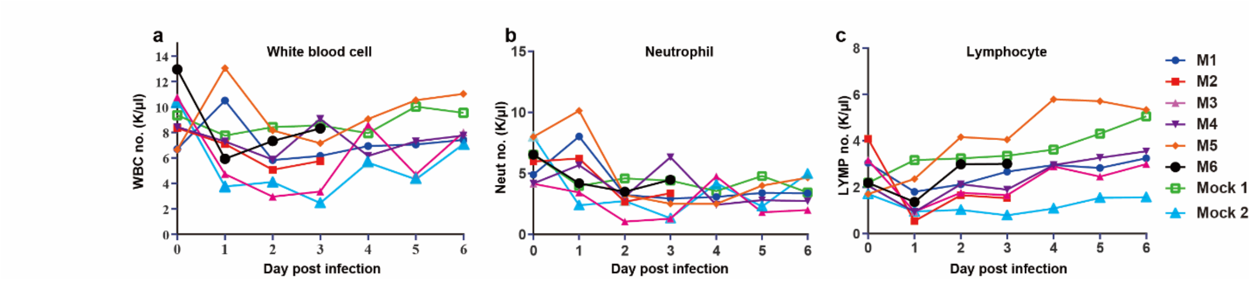

Supplementary information, Fig. S1: Hematological analysis in *Rhesus Macaques* inoculated with SARS-CoV-2. The counts of white blood cells (WBC) (a), Neutrophil count (b) and Lymphocyte (c) were analyzed.
