# Supplementary material for: Infection with novel coronavirus (SARS-CoV-2) causes pneumonia in Rhesus macaques
Source: Cell Res. 2020 Jul 7;30(8):670–7. doi: 10.1038/s41422-020-0364-z (PMC7364749; doi:10.1038/s41422-020-0364-z)
Supplement: Supplementary file 2 — Supplementary Figure S2 [file 41422_2020_364_MOESM2_ESM.pdf]

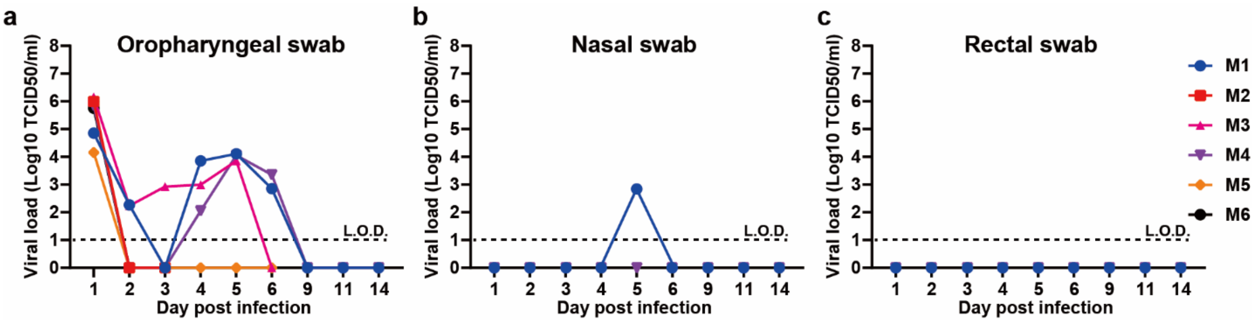

Supplementary information, Fig. S2 Virus in swabs. The supernatant from swabs were used for infectious virus detection on Vero cells. (a) Virus from oropharyngeal swabs. (b) Virus from nasal swabs. (3) Virus from rectal swabs. L.O.D.: limit of detection.
