# Supplementary material for: Infection with novel coronavirus (SARS-CoV-2) causes pneumonia in Rhesus macaques
Source: Cell Res. 2020 Jul 7;30(8):670–7. doi: 10.1038/s41422-020-0364-z (PMC7364749; doi:10.1038/s41422-020-0364-z)
Supplement: Supplementary file 3 — Supplementary Figure S3 [file 41422_2020_364_MOESM3_ESM.pdf]

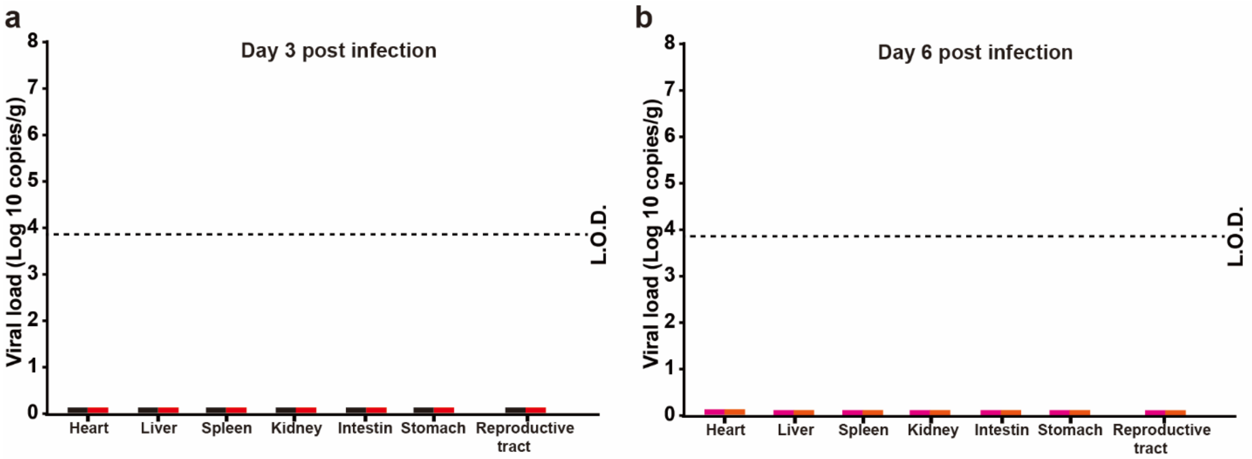

Supplementary information, Fig. S3 Viral loads in tissues collected from *Rhesus Macaques* infected with SARS-CoV-2. Four Rhesus Macaques were inoculated with SARS-CoV-2 and euthanized on day 3 and 6 post infection. Beside the tissue samples from respiratory tract, heart, liver, spleen, kidney, intestine, stomach and reproductive tract samples were collected as well. The one-step real time RT-PCR were performed to quantify the viral RNA from those tissues. (a) Viral RNA load in organ/tissues on day 3 post infection. (b) Viral RNA load in organ/tissues on day 6 post infection. L.O.D.: limit of detection.
