# Supplementary material for: Infection with novel coronavirus (SARS-CoV-2) causes pneumonia in Rhesus macaques
Source: Cell Res. 2020 Jul 7;30(8):670–7. doi: 10.1038/s41422-020-0364-z (PMC7364749; doi:10.1038/s41422-020-0364-z)
Supplement: Supplementary file 4 — Supplementary Figure S4 [file 41422_2020_364_MOESM4_ESM.pdf]

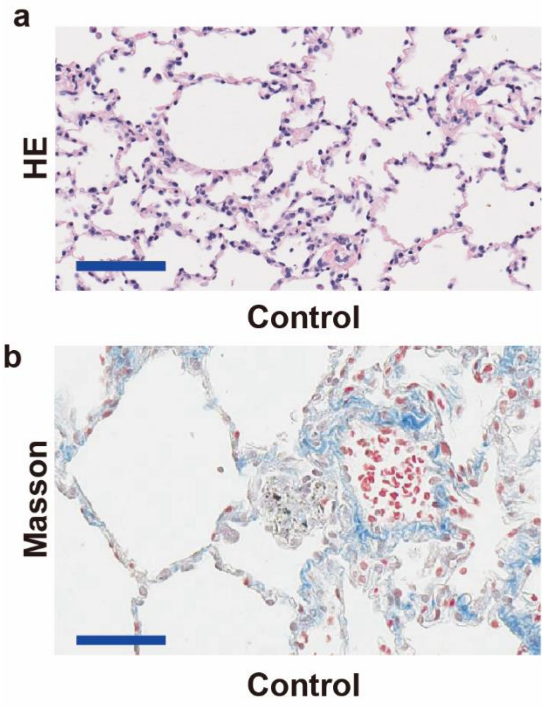

Supplementary information, Fig. S4 Histopathological analysis of lung changes in control rhesus macaques. Two RMs were inoculated with DMEM. Histological analysis was performed on tissues collected at 3 dpi and 6 dpi. (a) HE staining of the lung tissues. (b) Masson staining of lung tissue. Blue scale bar: 100 µm.
