# Supplementary material for: Infection with novel coronavirus (SARS-CoV-2) causes pneumonia in Rhesus macaques
Source: Cell Res. 2020 Jul 7;30(8):670–7. doi: 10.1038/s41422-020-0364-z (PMC7364749; doi:10.1038/s41422-020-0364-z)
Supplement: Supplementary file 5 — Supplementary Figure S5 [file 41422_2020_364_MOESM5_ESM.pdf]

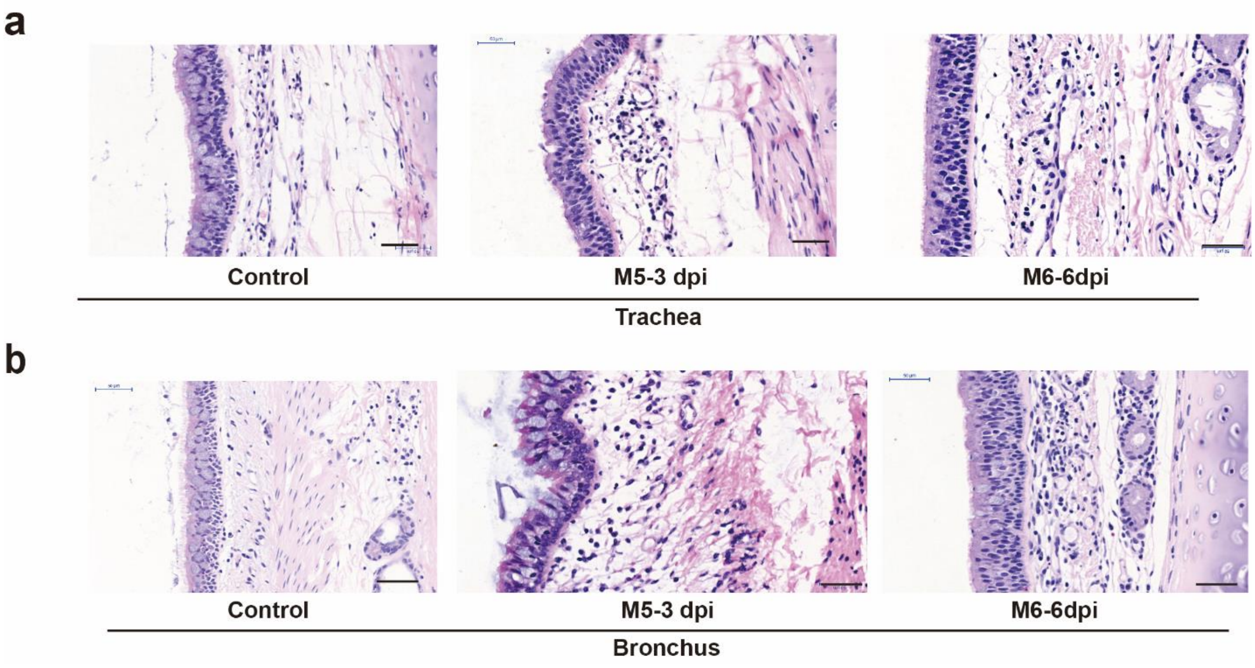

Supplementary information, Fig. S5 Histopathological analysis of trachea (a) and bronchus (b) changes collected on day 3 and 6 after inoculation. On day 3 post infection, the significant edematory epithelial cells of trachea and bronchus were observed with the brush border disappeared. The edema was also observed in the submucosal layer. Lymphocytes infiltration, eosinophils infiltration and degranulation were shown. On day 6 post infection, the histopathological changes of trachea were recovered. The edema of the bronchus epithelial cells, loss of brush border and submucosal layer were relieved, while the infiltration of eosinophils and mononuclear cells at submucosal layer still existed. The germinal center of the lymphoid tissue enlarged with multinucleated giant cells observed. Scale bar=50 µM.
