# Supplementary material for: Infection with novel coronavirus (SARS-CoV-2) causes pneumonia in Rhesus macaques
Source: Cell Res. 2020 Jul 7;30(8):670–7. doi: 10.1038/s41422-020-0364-z (PMC7364749; doi:10.1038/s41422-020-0364-z)
Supplement: Supplementary file 6 — Supplementary Figure S6 [file 41422_2020_364_MOESM6_ESM.pdf]

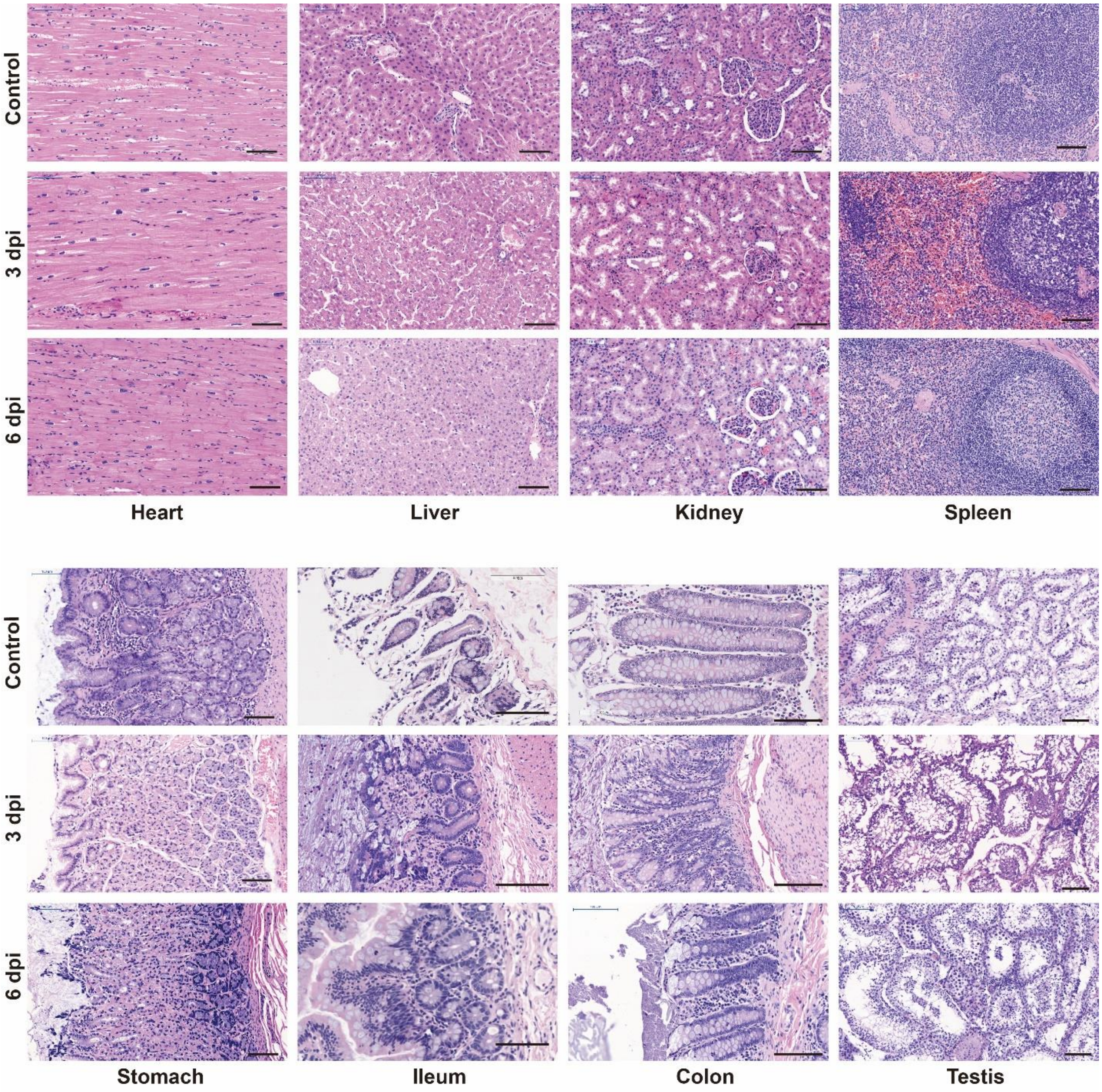

Supplementary information, Fig. S6 Histopathological changes of non-respiratory tract tissues.

Four Rhesus Macaques were inoculated with SARS-CoV-2 and euthanized on day 3 and 6 post infection. The tissues were collected on day 3 and 6 post infection and subjected for the histopathological analysis as described in material and methods. Scale bar=100 µM.
