# Supplementary material for: Infection with novel coronavirus (SARS-CoV-2) causes pneumonia in Rhesus macaques
Source: Cell Res. 2020 Jul 7;30(8):670–7. doi: 10.1038/s41422-020-0364-z (PMC7364749; doi:10.1038/s41422-020-0364-z)
Supplement: Supplementary file 7 — Supplementary Figure S7 [file 41422_2020_364_MOESM7_ESM.pdf]

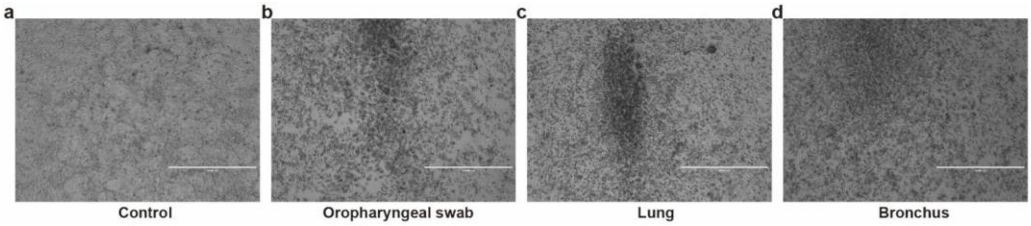

Supplementary information, Fig. S7 Cytopathic effect from cell culture. The tissues were collected on day 3 post infection. The supernatant was used to infect the Vero cell and re-isolate virus from tissues. On day 3 post infection, lots of dead cells which were floating in the medium were observed. The images from control (a), oropharyngeal swab (b), lung (c) and bronchus (d) infected wells were taken on 3 days post infection. Scale bar=1000 μM.
