# Supplementary material for: Infection with novel coronavirus (SARS-CoV-2) causes pneumonia in Rhesus macaques
Source: Cell Res. 2020 Jul 7;30(8):670–7. doi: 10.1038/s41422-020-0364-z (PMC7364749; doi:10.1038/s41422-020-0364-z)
Supplement: Supplementary file 8 — Supplementary Table S1 [file 41422_2020_364_MOESM8_ESM.pdf]

Supplementary information, Table S1 The rhesus macaque information used in the study

Group	ID	Age	Gender	Weight
C1	T094029	11	M	9.0
C2	T122528	8	F	5.3
RM1	T112703	9	M	11.2
RM2	T136008	7	F	8.2
RM3	T126106	8	F	6
RM4	T136106	7	F	7.8
RM5	T112511	9	M	11.3
RM6	T110003	9	M	11.5
